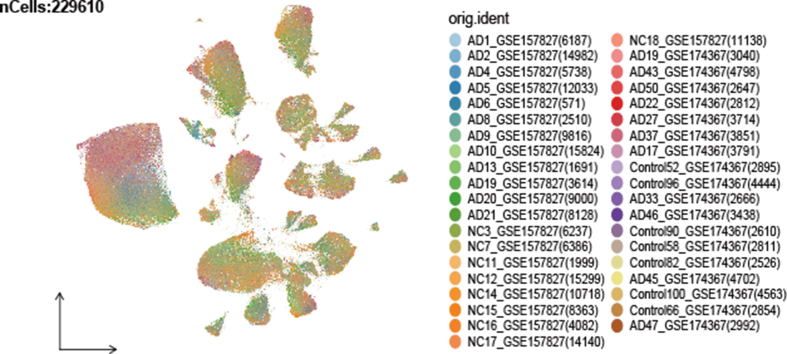# Identification and experimental validation of m7G-related molecular subtypes, immune signature, and feature genes in Alzheimer's disease

**DOI:** 10.1016/j.heliyon.2024.e33836

**Published:** 2024-07-01

**Authors:** Piaopiao Lian, Xing Cai, Cailin Wang, Heng Zhai, Ke Liu, Xiaoman Yang, Yi Wu, Zhuoran Ma, Xuebing Cao, Yan Xu

**Affiliations:** a Department of Neurology, Union Hospital, Tongji Medical College, Huazhong University of Science and Technology, Wuhan, China; b Department of Oncology, Union Hospital, Tongji Medical College, Huazhong University of Science and Technology, Wuhan, China; c Department of Neurology, Renmin Hospital of Wuhan University, Wuhan, China

**Keywords:** Alzheimer's disease, N7-methylguanosine, Bioinformatic analysis, Characteristic genes

## Abstract

**Background:**

Studies has shown that N7-methylguanosine (m7G) modification plays a critical role in neurological diseases. However, the exact role and association of m7G with the immune microenvironment in Alzheimer's disease (AD) remain largely unknown and unexplored.

**Methods:**

The study datasets comprised 667 AD samples and 503 control samples selected from eight datasets in the Gene Expression Omnibus database; m7G regulator genes were obtained from previous literature. The AD subtypes were identified by consensus clustering analysis according to m7G regulator genes. The clinical characteristics, immune infiltration, and biological functions of the AD subgroups were evaluated. A combination of different types of machine-learning algorithms were used for the identification of AD genes. We also assessed and validated the diagnostic performance of the identified genes via qRT-PCR, immunofluorescence, and immunohistochemical analyses.

**Results:**

Two AD distinct subgroups, namely cluster A and cluster B, were identified. Cluster A had poor pathological progression and immune infiltration, representing a high-risk subgroup for AD. The differentially expressed genes of cluster A were enriched in immune and synapse-related pathways, suggesting that these genes probably contribute to AD progression by regulating immune-related pathways. Additionally, five feature genes (*AEBP1, CARTPT, AK5, NPTX2*, and *COPG2IT1*) were identified, which were used to construct a nomogram model with good ability to predict AD. The animal experiment analyses further confirmed that these feature genes were associated with AD development.

**Conclusion:**

To the best of our knowledge, this is the first study to reveal close correlations among m7G RNA modification, the immune microenvironment, and the pathogenesis of AD. We also identified five feature genes associated with AD, further contributing to our understanding of the underlying mechanisms and potential therapeutic targets for AD.

## 1 Background

As the most common cause of dementia, Alzheimer's disease (AD) affects >50 million people globally [1]. The incidence of AD has been steadily increasing, placing a continuous burden on the global community [2]. AD pathology is primarily characterized by amyloid-beta (Aβ) and neurofibrillary tangles (NFTs) deposits [3], and clinically manifests as progressive memory impairment. However, the underlying mechanisms of AD remain elusive, and current treatments solely alleviate symptoms without changing the course of the disease [4,5]. Hence, investigating the underlying mechanisms of AD progression is crucial. Additionally, classifying patients with AD not only facilitates the development of individualized treatment and management strategies but also enables more accurate prediction of disease progression, thereby improving treatment outcomes and the quality of life.

RNA modifications affect various RNA processes, such as generation, transport, function, and metabolism, which are considered indispensable regulators of cell biology [6,7]. To date, over 170 types of RNA modifications have been identified [8]. N7-methylguanosine (m7G) is a recently reported mRNA 5′ cap modification that plays an important role in mRNA export, translation and splicing [9,10]. Additionally, m7G methylation can also occur in tRNA [11], rRNA and microRNA [12,13]. Recent research has highlighted the crucial role of m7G methylation in the nervous system and its strong association with the onset and development of various brain diseases [14], including microcephalic primordial dwarfism [15,16], Down syndrome, ischemic stroke or hypoxia [17], and multiple sclerosis [18]. A recent study demonstrated that overexpression of the m7G methyltransferase Mettl1, which is responsible for m7G methylation of internal mRNAs, ameliorated neurogenesis and behavioral defects in APP/PS1 transgenic mice [19]. This finding highlights the critical role of m7G methylation in AD progression. However, only few studies have explored the role and mechanisms of m7G methylation in AD. With the development of sequencing technologies, more and more samples are converted into useable data information. Publicly available datasets for AD are also becoming more readily available. Consequently, our objective was to investigate the potential role of m7G in AD using bioinformatics data mining.

Contrary to previous studies that focused only on one or two m7G regulator genes, in this study, we obtained a total of 30 m7G regulator genes from the literature and compared their differential expression between patients with AD and healthy individuals. Based on differentially expressed m7G-related genes, our research identified two m7G-related subgroups with different m7G scores; moreover, we compared the differences in the clinical characteristics, immune infiltration signatures, and biological functions between the subgroups. Furthermore, using a cross-combination of three machine-learning algorithms, including least absolute shrinkage and selection operator (LASSO) regression, Random Forest (RF), and support vector machine-recursive feature elimination (SVM-RFE), we identified five feature genes associated with AD. This five-gene diagnostic signature demonstrated excellent discriminatory ability in identifying patients with AD and represents a promising set of robust biomarkers and potential AD therapeutic targets.

## 2 Methods

### 2.1 Dataset source

Expression profiles and clinical details of patients with AD were downloaded from the GEO database [20]. We downloaded the following eight datasets: GSE106241, GSE122063 [21], GSE118553, GSE132903 [22], GSE5281 [23], GSE48350 [24], GSE28146 [25], and GSE8442201 (GSE8442201 originates from the annotations of the GPL570 platform within GSE84422). Given that the cerebellum is often considered to be partially spared from AD because of the general absence of reported plaques and tangles, we excluded the cerebellum samples of GSE118553. A comprehensive description of all datasets is presented in Table S1. Considering batch effects and data quality, we implemented a series of interventions on the dataset, including data cleaning and application of “Combat” algorithm to remove batch effects among the datasets. Finally, a merged dataset that included information on 667 AD samples and 503 normal samples was obtained.

### 2.2 scRNA-seq data analysis

Furthermore, we integrated 23 AD samples with scRNA-seq data and 16 normal samples with scRNA-seq data from GSE174367 [26] and GSE157827 [27]. We analyzed the scRNA-sequencing data by the “Seurat” package. To ensure the quality of data, cells exhibiting nFeature <300 or >10,000, nCount >100000, and percent.mt >10 % were excluded. Subsequently, we normalized the scRNA-seq data using the “NormalizeData” function. Following the initial preprocessing steps, 3000 hypervariable genes were identified by “vst” methods. The number of principal components was estimated using “RunPCA” function, and the main principal components were summarized using uniform manifold approximation and projection (UMAP) approximation analysis. The Harmony method was used to correct for batch effects.

### 2.3 Identification of m7G-related subtypes

m7G regulator genes were acquired from previous studies [28,29]. Table S2 provides detailed gene information. We compared the differential expression of m7G-related genes between patients with AD and healthy individuals. Subsequently, we used the R package “corrplot” to assess the Spearman's rank correlations between the m7G genes. To classify AD patients into distinct molecular classification, we performed consensus clustering based on the m7G regulator genes [30]. The maximum of clusters was set at 10 (the group consensus score threshold >0.8). We used the gene-set variation analysis method to calculate the m7G score of each sample.

### 2.4 Analysis of the clinical characteristics of m7G-related subtypes

Wilcoxon's rank-sum test (conducted pairwise) was used to compare the differences in α-, β-, and γ-secretases; Braak stages; NFT densities, and age between the two m7G subgroups. Furthermore, the proportions of tissue, sex, and *APOE4* alleles in the two groups were also calculated.

### 2.5 Evaluation of immune infiltration in m7G-related subgroups

The following algorithms in “IOBR” package were used to evaluate the levels of immune infiltration: EPIC [31], IPS [32], quanTIseq [33], CIBERSORT [34], TIMER [35], XCELL [36], MCPCounter [36], and ESTIMATE [37]. Based on transcriptomic data, these algorithms were able to quantify the absolute abundance of 24 immune cells in each sample. Furthermore, we also applied gene-set enrichment analysis (ssGSEA) to calculate the enrichment score [38].

### 2.6 Recognition of differentially expressed genes (DEGs) and functional enrichment analysis

The DEGs between AD and normal samples within the two m7G-related subgroups were identified based on an absolute logFC value of >0.35 and an adjusted *P*-value of <0.05. To further investigate the functions of m7G-related DEGs, we used “clusterProfiler” [39]. The R package was used to perform GO and KEGG analyses on the DEGs. Additionally, we performed GSEA to further explore special functions of clusters A and B.

### 2.7 Selection of AD feature genes

Considered together, the results of abovementioned analyses suggest that cluster A may be a high-risk subtype for AD; thus, we conducted a further detailed investigation on the DEGs of cluster A. To narrow the scope of feature genes, DEGs that met the criteria of an absolute logFC of >1 and an adjusted *P*-value of <0.05 in cluster A were employed for machine-learning analysis. Each algorithm possesses distinct strengths and weaknesses, and the applicability and effectiveness of machine-learning algorithms for biomarker screening have improved in recent years. We adopted three machine-learning algorithms—LASSO regression, RF, and SVM-RFE—to identify the feature genes of AD. The LASSO regression algorithm in the “glmnet” R package was used to identify the feature genes associated with the discrimination of AD and normal individuals [40]. RF consists of multiple decision trees, analogous to a forest with many trees, and has demonstrated superior accuracy compared to other methods. Furthermore, SVM-RFE algorithm is most frequently applied in various algorithm [41]. To prevent overfitting, these machine-learning methods are based on a 10-fold cross-validation approach. Cross-validation was performed using the respective built-in function. Thus, we intersect the results of the three algorithms to screen feature genes.

### 2.8 Construction and validation of a diagnostic model for AD

Training set and testing set were randomly assigned from the previously merged datasets. We defined the predictor variables for AD as the identified feature genes and constructed a nomogram model in training set. The goal of diagnostic model is to diagnosis outcomes as accurately as possible. Therefore, we applied calibration curves, DCA analysis, and AUC values to evaluate the diagnostic model in training and testing sets.

Furthermore, we calculated the AUC values by “pROC” package in six independent datasets to examine whether the model could be generalized.

### 2.9 Animals

The P301S transgenic mice used in this study were obtained as a gift form Professor Gang Li at the Wuhan Union Hospital [42]. We used six 8-month-old P301S male mice as the AD group and six age-matched male C57BL/6J mice as the control group. Throughout the experiment, a cage of three mice was kept in a standard experimental environment with free access to food and water. All animal experiments were reviewed and approved by the Ethics Committee of Tongji Medical College, Huazhong University of Science and Technology.

### 2.10 qRT-PCR assay

Half of the mice in each group were randomly selected and anesthetized. The RNA samples of the cortical tissue of the mice in each group were obtained by using total RNA isolation kit (Takara, Japan). Subsequently, we used the reverse-transcription kit to reverse transcribed RNA to cDNA. According to the manufacturer's guideline, we combined cDNA, ChamQ SYBR qPCR Master Mix (Vazyme, China), and primers in a reaction and measured the mRNA levels of the feature genes using StepOnePlus real-time PCR System. All primers applied for RT-PCR analysis are listed in Table S3.

### 2.11 Immunofluorescence (IF) analysis

The remaining mice were anesthetized and perfused with sodium and 4 % ice-cold paraformaldehyde; subsequently, their brains were removed and immersed in 4 % paraformaldehyde. The fixed brain specimens were embedded in paraffin, cut coronally into 5-μm sections, and mounted on slides. Then, the slides were dewaxed with xylene, rehydrated with ethanol, water bathed in citrate solution for antigen retrieval and washed with phosphate-buffered saline thrice. The slides were blocked in 3 % bovine serum albumin and incubated with anti-AEBP1 antibody (1:100, Abclonal, A16340) overnight at 4 °C. Subsequently, the slides were incubated with secondary antibodies for 50 min in a light-free environment. Next, we incubated the slides with a DAPI solution. Images were acquired using a digital slice scanning system (CaseViewer).

### 2.12 Immunohistochemistry (IHC) analysis

The same protocol as the IF staining, up until the incubation with the secondary antibody, was used to perform IHC staining. Primary antibodies recognizing CARTPT (1:100, Abclonal, A18275) were used for the IHC staining. Subsequently, the sections were washed and incubated with rabbit antibody at a temperature of 25 °C for 1 h. Following another round of washing, the sections were subjected to DAB and counterstained with hematoxylin. Finally, the sections were sealed. Images were acquired using a digital slice scanning system (CaseViewer).

### 2.13 Statistical analyses

R (version 4.2.1) was used for all statistical analyses. Between-group comparisons were performed using the Wilcoxon test, and a *P*-value of less than 0.05 was considered statistically significant.

## 3 Results

### 3.1 Differential expression of m7G regulator genes in ADs and non-ADs

Fig. 1 represented the flow of the entire research strategy (Fig. 1). We collected 30 m7G regulator genes from previous studies [28,29] and are shown in Table S2. The expression analysis identified 24 of the 30 m7G RNA regulator genes in human brain samples (Fig. 2A). Among them, 15 m7G regulator genes exhibited marked differential expression in brain tissues, with six of them (*CYFIP1*, *LSM1*, *NCBP2*, *NUDT16*, *NUDT3*, and *QKI*) being upregulated in patients with AD, and nine of them (*DCPS, LARP1, METTL1, NUDT10, EIF4E, EIF4E2, EIF4E3, NUDT11*, and *SNUPN*) were downregulated compared to nonAD samples. Additionally, we conducted Pearson correlation analysis to explore the relationships among the expression patterns of the 24 m7G regulator genes in the AD datasets (Fig. 2B). The results showed that the 24 m7G regulator genes had a close connection, establishing the groundwork for subsequent m7G cluster analysis. NUDT10 and NUDT11 were noted to be the most closely associated regulators in terms of expression, implying a collaborative functional relationship between them.

**Fig. 1:**
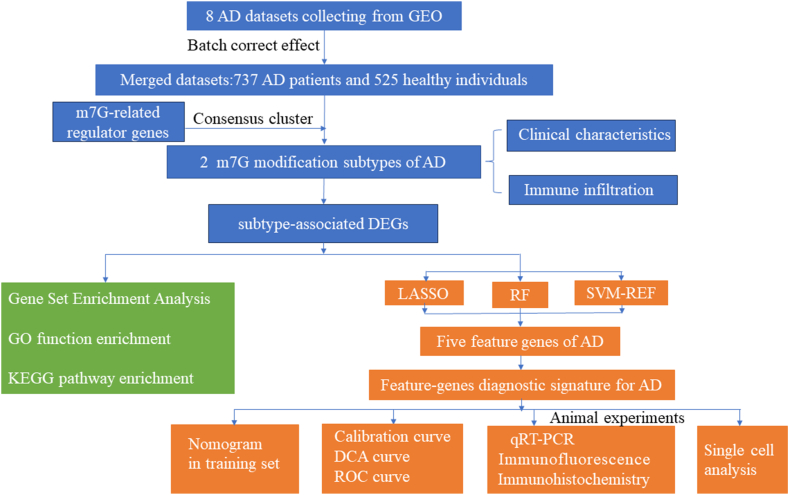
The study flowchart.

**Fig. 2:**
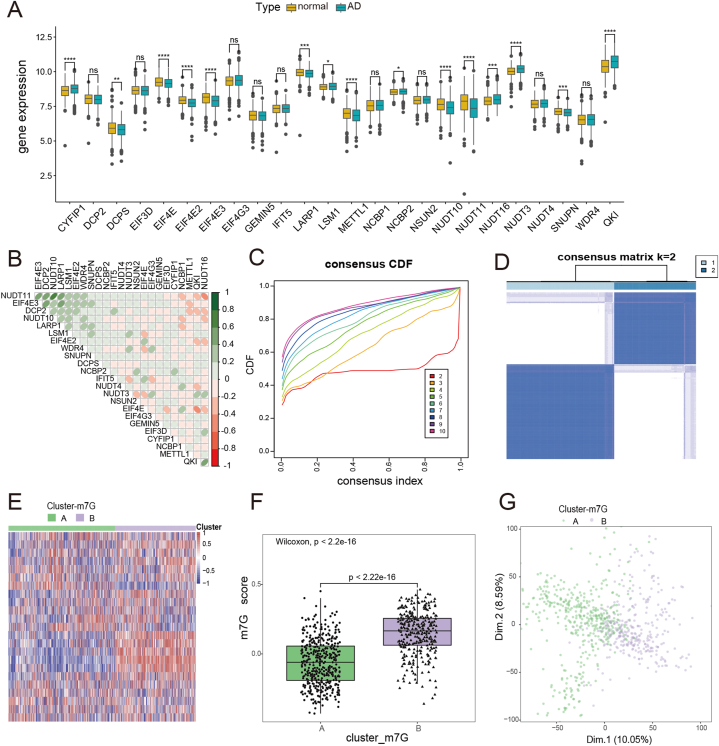
Identification of m7G-related subtypes based on the expression of m7G regulator genes. (A) Expression profiles of m7G regulators genes between AD patients and NCs. ns = no significant, *p < 0.05, **p < 0.01, ***p < 0.001, and ****p < 0.001 vs. the NC group. (B) Pearson correlation analysis of 24 m7G regulator genes expressions in ADs. (C) Representative consensus CDF curve when k = 2–10. (D) Consensus clustering matrix when k = 2. The two subtypes were named as Cluster A and Cluster B (E) Heatmap showing the expression of m7G regulators genes in two subtypes. (F) The m7G-score in two subtypes. (G) PCA showing a significant difference between the two subtypes.

### 3.2 Consensus clustering analysis of distinct subtypes in AD

Except for the cerebellar region, we noted no significant anatomical differences in other brain regions (Fig. S1), which is consistent with previous reports [43]. Therefore, we only adjusted for batch effects caused by different platforms to avoid excessive adjustment. The principal component analysis (PCA) plot showing successful removal of batch effects (Fig. S2). Consensus clustering analysis classified 743 AD samples into different groups according to the m7G regulator genes [44]. We classified these samples into 2 to 10 subgroups (Fig. S3). Final subtypes were determined based on the consensus clustering matrix and cumulative distribution function (CDF) plots. According to the consensus matrix and CDF plots, we chose k = 2 as the most favorable value to categorize patients with AD into two different subtypes (Fig. 2C and D), namely cluster A and cluster B. Both PCA and a heatmap of m7G-related gene expression further confirmed apparent difference between the two subtypes (Fig. 2E, G). In addition, we gave each patient a corresponding m7G score based on the composite score of m7G regulator gene expression in patients with AD. We compared the scores of the two subtypes. The results showed that the m7G score was higher for cluster B than that for cluster A (*p* < 0.001) (Fig. 3F).

**Fig. 3:**
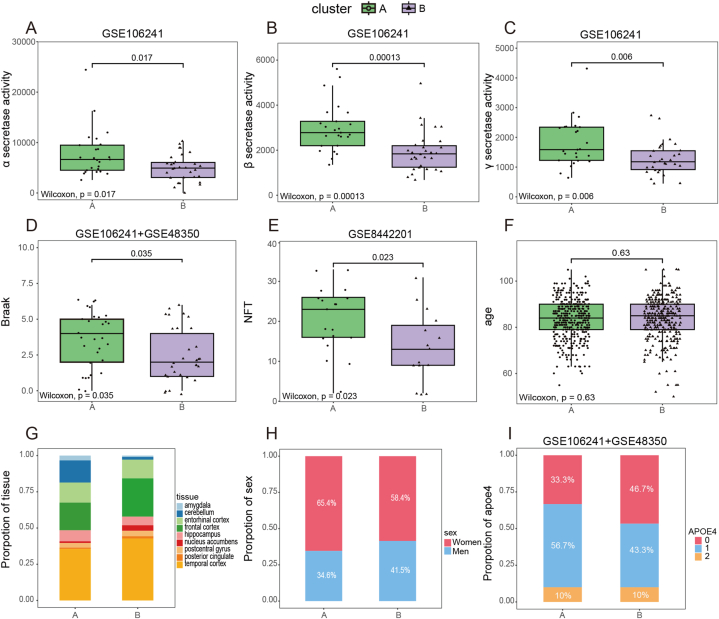
Clinical characteristics analysis of m7G-related subtypes. (A) Comparison of α-secretase activity, (B) β-secretase activity, (C) γ-secretase activity, (D) Braak, (E) NFT, and (F) age between two subgroups. (G) The proportion of tissues, (H) sex, and (I) APOE 4 alleles of each subgroup.

### 3.3 Comparison of clinical characteristics between the two m7G subtypes

In cluster A, the enzymatic activities of α-, β-, and γ-secretases are elevated compared to those in cluster B (Fig. 3A–C), especially β-secretases activities (*p* < 0.001) and γ-secretases activities (*p* < 0.01). Aβ plaques are generated as a result of the enzymatic cleavage of amyloid precursor protein by the β and γ secretases successively [45]. Additionally, the NFT density (*p* < 0.05) and Braak stage (*p* < 0.05) were also higher in cluster A than in cluster B (Fig. 3D and E). However, no statistical difference was observed in the age between the two groups (Fig. 3F). The tissue origins of clusters A and B was shown in Fig. 3G. Braak stage is based on the extent and distribution of tau NFTs, with more advanced staging indicating a higher tangle burden. In both subgroups, the proportion of women exceeded that of men, and similarly, the proportion of people with APOE4 allele exceeded that of people without APOE4 allele (Fig. 3H and I).

### 3.4 Comparison of immune signatures between the two m7G subgroups

To further explore the immune signatures of two m7G subtypes, ESTIMATE was used to calculate stromal and immune cells scores. The results revealed a notable difference in both stromal and immune cells scores between the two groups. Cluster A exhibited higher stromal and immune scores than in cluster B (*p* < 0.001) (Fig. 4B). To better elucidate the immunological distinctions, we further compared the variations in 24 immune cells within each subtype. The varying infiltration degrees of the 24 immune cell types in the samples as well as the expression levels of the immune checkpoints are demonstrated in the heatmap (Fig. 4A). We observed higher expression of multiple immune checkpoints in cluster A, including TLR9, CTLA4 and TNFRSF9, which may be targets for immunotherapy (Fig. 4C). Additionally, as shown in Fig. 4B, the proportion of CD4^+^ T cells (*p* < 0.001); regulatory T cells (*p* < 0.001); memory CD4^+^ T cells (*p* < 0.001); natural B cells (*p* < 0.01); memory B cells (*p* < 0.001); activated NK cells (*p* < 0.05); resting NK cells (*p* < 0.005); resting mast cells (*p* < 0.005); activated mast cells (*p* < 0.001); M0, M1, and M2 macrophages (*p* < 0.001); monocyte (*p* < 0.01); resting dendritic cells (*p* < 0.05); activated dendritic cells (*p* < 0.001); neutrophils (*p* < 0.001), endothelial cells (*p* < 0.001); and fibroblasts (*p* < 0.001) were significant higher in cluster A than in cluster B (Fig. 4D). We also quantified different types of CAFs. The results showed that nearly all subtypes of fibroblasts were enriched in cluster A, and normal fibroblasts were lacking (Fig. 4E). The comprehensive overview of the immune infiltration assessment performed using multiple algorithms is presented in Fig. S3.

**Fig. 4:**
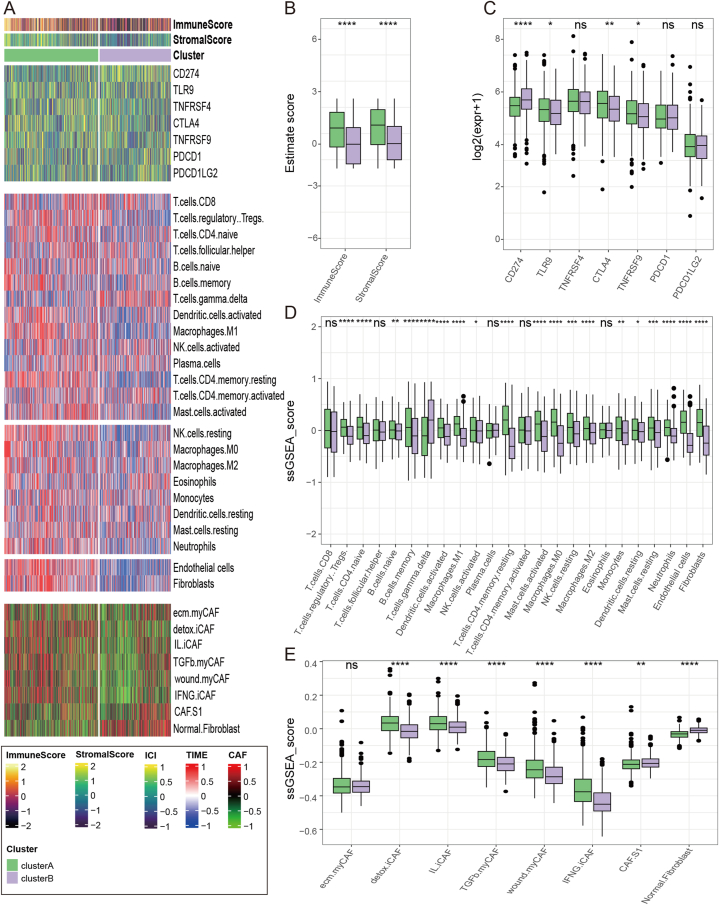
Immune signature between two m7G subtypes. (A) Heatmap showing the overall immune infiltration signature in two m7G subtypes. Boxplot pictures showing the differences of (B) immune and stromal score, (C) immune checkpoint targets, (D) infiltrated immune cells, and (E) fibroblast signatures between two AD subgroups. (ns indicates no significance, *p < 0.05, **p < 0.01, ***p < 0.005, ****p < 0.0001).

### 3.5 DEGs and functional enrichment analysis

**Fig. 5:**
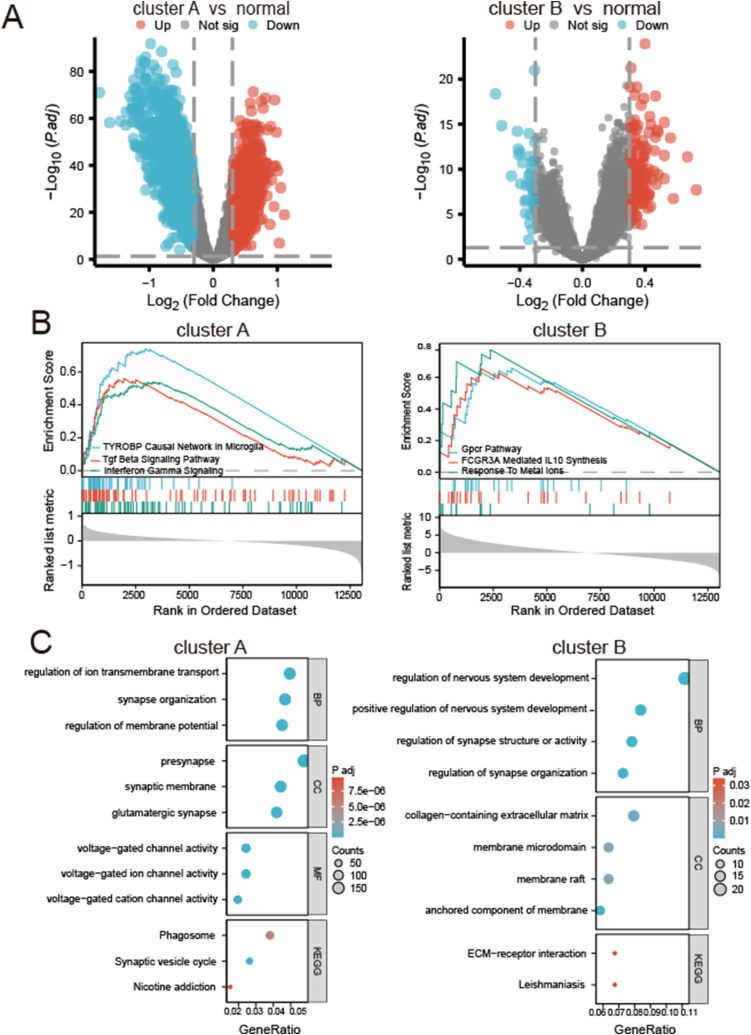
Enrichment analysis of DEGs in AD. (A) Differentially expressed genes (DEGs) between AD and normal samples in cluster A and cluster B. Results of (B) GSEA, (C) KEGG, and GO enrichment analysis of DEGs in cluster A and cluster B.

The number of DEGs between AD and normal samples in clusters A and B were 3254 and 195, respectively (Fig. 5A). GSEA was used to explore the potential functions of DEGs (Table S4). As shown in Fig. 5B, genes highly expressed in AD patients of cluster A were significantly enriched in several hallmark gene sets, including TYROBP causal network in microglia, TGF-β signaling pathway, and INF-γ signaling gene sets. The genes highly expressed in AD patients of cluster B were significantly enriched in several hallmark gene sets, including G-protein-coupled receptor pathway, FCGR3A-mediated IL10 synthesis, and response to metal ions gene sets. Furthermore, we conducted GO and KEGG enrichment analyses according to the identified DEGs (Table S5, Fig. 5C). GO enrichment showed that the biological process (BP) in cluster A mainly involved regulation of ion transmembrane transport, synapse organization, and regulation of membrane potential. The BP in cluster B was mainly related to regulation of nervous system development and synapse structure or activity. According to KEGG pathway analysis, cluster A mainly participated in the phagosome, synaptic vesicle cycle, and nicotine addiction pathway, whereas cluster B was primarily participated in extracellular matrix–receptor interaction and the Leishmaniasis pathway.

### 3.6 Selection of AD feature genes

**Fig. 6:**
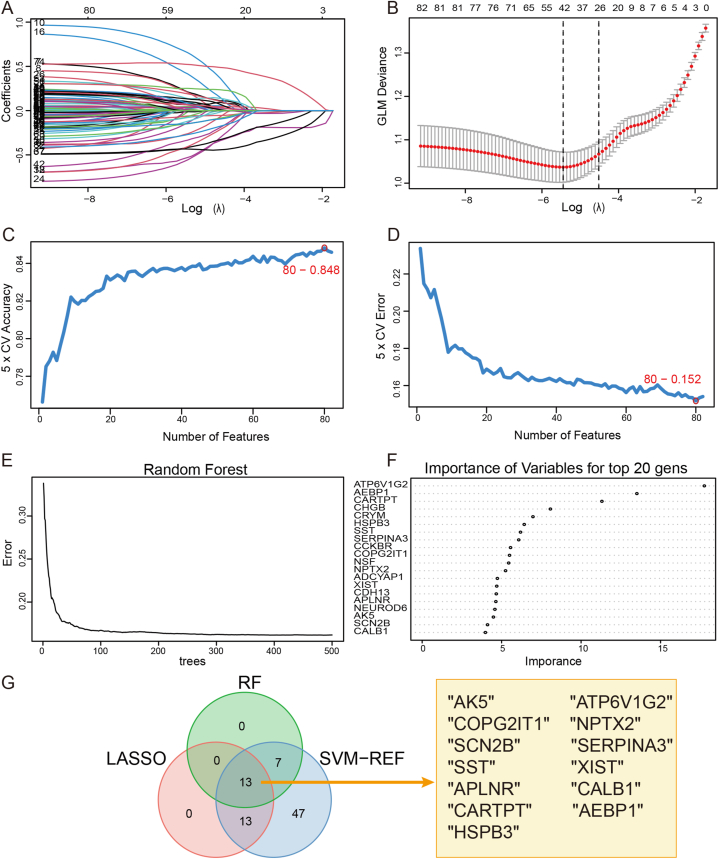
Selection of the AD feature genes by machine-learning algorithms. (A, B) LASSO regression, (C, D) SVM-REF, and (E, F) RF to identify AD feature genes. (G) Representative Venn diagram shows overlapping feature genes.

First, we identified 82 DEGs within cluster A with an absolute logFC of >1 and adj *P*-value of <0.05. Second, we performed three algorithms to further identify AD biomarkers based on the above DEGs. We identified a subset of 26 characterized genes by the LASSO regression (Fig. 6A and B). A subset of 82 feature genes was identified using the SVM-RFE algorithm, (Fig. 6C and D). The RF algorithm screened genes, displaying the top 20 feature genes (Fig. 6E and F). A total of 13 genes overlapped across three algorithms. (*AK5, ATP6V1G2, COPG2IT1, NPTX2, SCN2B, SERPINA3, SST, XIST, APLNR, CALB1, CARTPT, AEBP1, and HSPB3*) (Fig. 6G). Eventually, we performed logistic regression to select five feature genes (*AEBP1*, *CARTPT, AK5, NPTX2,* and *COPG2IT1*; *p* < 0.05) from the 13 genes.

### 3.7 Construction and validation of a diagnostic model for AD

**Fig. 7:**
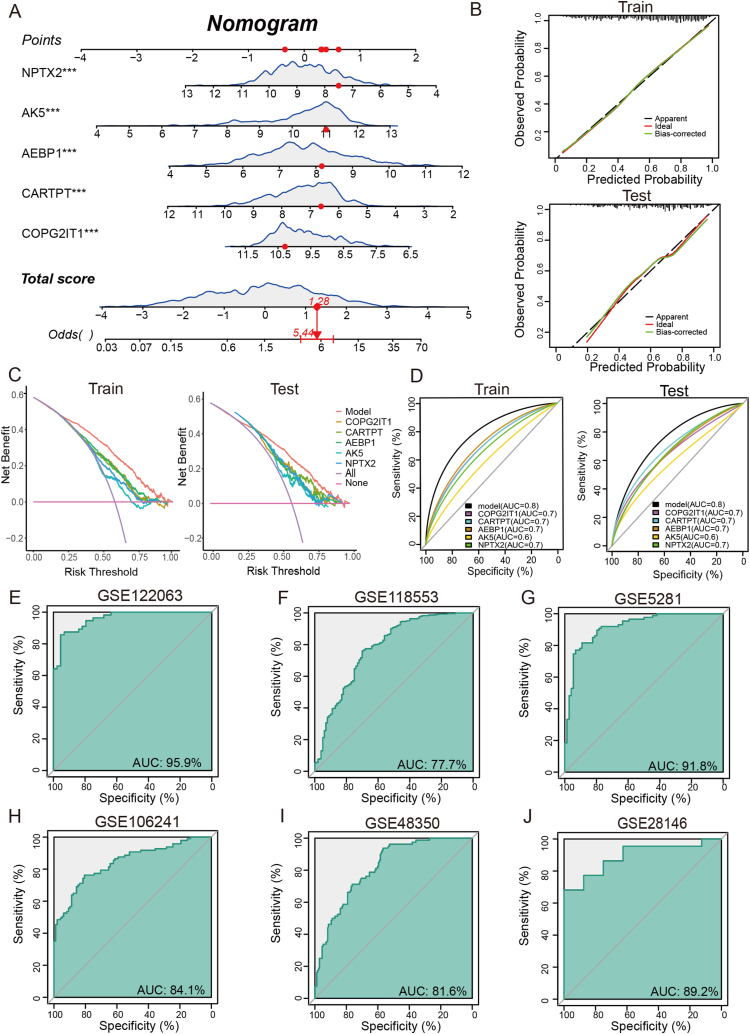
Construction and validation of a diagnostic AD model. (A) Nomogram of five-genes diagnostic model for AD. (B) Calibration curve evaluates prediction efficacy of the model. (C) DCA evaluates the clinical benefits of the model. (D) ROC curves evaluate the diagnostic efficacy of five feature genes in testing and training set. (E–L) ROC curves were also used to evaluate the diagnostic efficacy of feature genes in six single datasets including GSE122063 (E), GSE118553 (F), GSE5281 (G), GSE106241 (H), GSE48350 (I), and GSE28146 (J). (*p < 0.05, **p < 0.01, ***p < 0.005, ****p < 0.0001).

A nomogram model using five feature genes (*AEBP1*, *CARTPT, AK5, NPTX2,* and *COPG2IT1*) was developed for AD diagnosis (Fig. 7A). The calibration plot revealed minor error between predicted and actual probabilities from the nomogram, indicating high accuracy of the model (Fig. 7B). The DCA plot showed that the benefit of “model” curve was highest in all curves, suggesting that AD patients may benefit from the model (Fig. 7C). The ROC plot indicated that the AUC values of the nomogram model was 0.8 for both the training and testing sets, suggesting that the model had high discrimination between AD and normal individuals (Fig. 7D). Additionally, the AUC values for the model in each of the six separate datasets were 0.959 (GSE122063), 0.777 (GSE118553), 0.918 (GSE5281), 0.841 (GSE106241), 0.816 (GSE48350), and 0.892 (GSE28146) (Fig. 7E–L). All of the above assessments indicate that the five genes may be involved in AD progression.

### 3.8 Association between m7G-related genes and the five feature genes

**Fig. 8:**
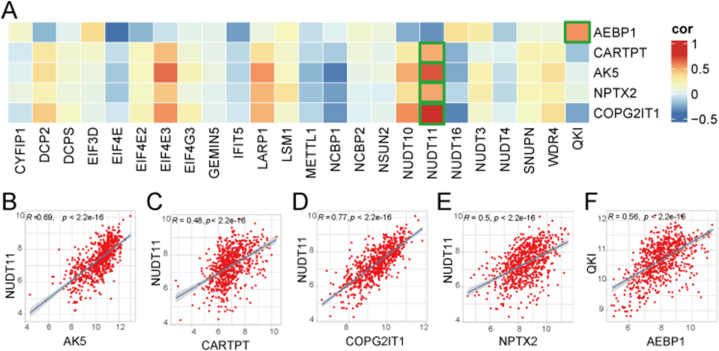
Relationship between m7G-related genes expression levels and five feature genes expression levels in AD patients. (A) The heatmap of correlation coefficient. (B–F) These scatter plots displayed the most positively correlated feature gens-m7G regulator pair.

To further elucidate the association between the feature genes and m7G-related genes in AD, Pearson correlation analysis was performed to analyze the correlation between the mRNA expressions of feature genes and m7G-related genes in patients with AD (Fig. 8A). The expression of NUDT11 was most positively correlated with the expression of four feature genes, including *AK5*, *CARTPT*, *COPG2IT1*, and *NPTX2* in patients with AD, with correlation coefficients of 0.69, 0.48, 0.77, and 0.5, respectively (Fig. 8B–E). The expression of AEBP1 was most positively correlated with that of QKI, with a correlation coefficient of 0.56 (Fig. 8F).

### 3.9 Differential expressions of the feature genes

**Fig. 9:**
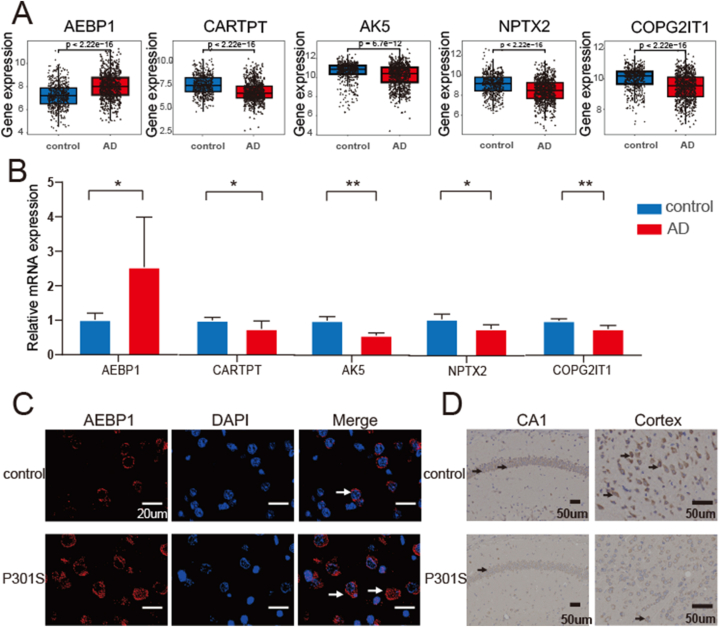
Different Expression between AD and control groups of the five feature genes. (A) Differential expression of the five genes in merged GEO dataset. (A) Validation of the expression of the five genes (AEBP1, CARTPT, NPTX2, COPG2IT1, and AK5) by quantitative real-time reverse-transcription PCR (qRT-PCR) using brain tissues from AD mice and controls. (*p < 0.05, **p < 0.01, ***p < 0.005, ****p < 0.0001). (C) Representative immunofluorescence images of AEBP1 in the cortices of AD and control group mice (scar bar: 20 μm). (D) Representative immunohistochemical images of CARTPT in the brains of AD and control group mice (scar bar: 50 μm).

**Fig. 10:**
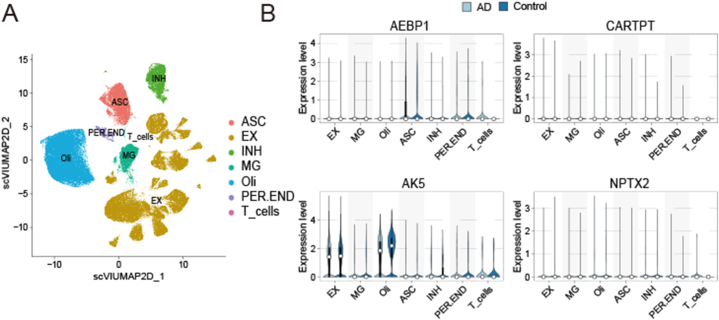
Expression of the five feature genes in different cell types in the scRNA-seq data. (**A**) UMAP displayed seven cell types. (**B**) The violin pictures showed the gene expression levels of AEBP1, CARTPT, AK5, and NPTX2 in different cell subgroups, split by control and AD samples.

First, we explored the expression of the feature genes between AD and normal brain samples in merged dataset. The analysis of mRNA expression showed that *AEBP1* was upregulated in the AD patients, whereas *AK5*, *COPG2IT1*, *CARTPT*, and *NPTX2* were downregulated in AD patients (Fig. 9A). Moreover, we also verified the expression of feature genes in AD mice. The results of mRNA analysis in AD mice were highly consistent with the results of bioinformatics analysis results (Fig. 9B). In addition to the qPCR method, we also used IF and IHC methods to validate the expression of *AEBP1* and *CARTPT*, as they exhibited the highest AUC values among the featured genes (Fig. 9C and D). The IF and IHC results further confirmed *AEBP1* was significantly upregulated and *CARTPT* was downregulated in the AD mice. To explore the cell-specific expression of the feature genes, we combined scRNA-seq data to analyze them. Furthermore, we performed UMAP dimensionality reduction and Leiden clustering on the batch-corrected transcriptomic datasets (Fig. S1); seven cell clusters were identified (Fig. 10A). The violin diagram illustrates the expression of the feature genes in seven cell types. *AK5* was annotated in all seven cell types; *AEBP1* was annotated in astrocytes, pericytes/endothelial cells, and T cells; and *CARTPT* and *NPTX2* were rarely annotated (Fig. 10B). *COPG2IT1* was not annotated in any of the seven cell types.

## 4 Discussion

AD is a progressive neurodegenerative disease that primarily affects memory and thinking [46,47]. It is characterized by the formation of amyloid plaques and tau tangles in the brain, leading to gradual loss of cognitive function. Despite decades of research, the pathogenesis of AD has not yet been fully understood and there is still a lack of effective treatment [48]. Therefore, early diagnosis and intervention for AD patients is essential. However, diagnosing AD has historically been challenging, and current biomarkers for AD require further supplementation. Identifying AD subtypes can help identify patient heterogeneity and drive advances in individualized targeted therapies for AD.

Recent studies have found that abnormal DNA modification, RNA modifications, histone modifications, noncoding RNAs, and other epigenetic modifications are closely associated with the development of AD [[49], [50], [51], [52]]. Among these, RNA methylations are believed to play a significant role in the onset and progression of diseases [53]. One of the common base modifications in post-transcriptional regulation, m7G, is also actively involved in a variety of physiological and pathological functions in the human body. Several studies have shown that m7G methylation is involved in the pathogenesis of various brain diseases [14], including AD. However, little is known about the role of m7G methylation in AD. Therefore, we aimed to explore the potential role of m7G in AD through bioinformatic methods.

First, we obtained 30 m7G regulator genes from previous studies and showed significant dysregulation of 15 of these genes in patients with AD. Most m7G regulator genes were downregulated in patients with AD compared with healthy individuals (*DCPS*, *EIF4E*, *EIF4E2*, *EIF4E3*, *LARP1*, *METTL1*, *NUDT10*, *NUDT11*, and *SNUPN*). This finding aligns with the results of a recent study where METTL1 was downregulated in AD mice [19]. The Pearson correlation analysis of 24 m7G regulators genes in the AD datasets suggested that *NUDT10* and *NUDT11* act synergistically in AD progression.

Based on these differentially expressed m7G genes, we identified two distinct AD subgroups: cluster A and cluster B. Compared with cluster B, cluster A exhibited a lower m7G score, suggesting reduced expression of m7G regulator genes. Compared with the patients in cluster B, those in cluster A showed more severe AD neuropathological signatures (higher β- and γ-secretase activities, Braak stage, and NFT densities) and higher expression of *APOE4*, an AD genetic risk factor. We hypothesized that decreased m7G methylation is a risk factor in AD progression. In addition to *METTL1*, the other methylated genes in the abovementioned results that are downregulated in patients with AD have not been studied, and targeting these genes may be a potential therapeutic option for AD.

The progression of AD is closely linked to immune and inflammatory activation [54,55]. There is evidence suggesting that m7G modifications are associated with neuroinflammation in AD. CD47, a receptor of the immunoglobulin superfamily, interacts with exportin-1 to regulate m7G-modified miRNAs and mRNAs (e.g., let-7) in extracellular vesicles [12,56]. The let-7 family triggers intracellular signaling that leads to neuroinflammation and central nervous system neurodegeneration [57]. In immune infiltration analysis, cluster A was accompanied by significantly more immune cell infiltration than cluster B. These cell populations mainly included B cells, T cells, macrophages, NK cells, mast cells, dendritic cells, and neutrophils, suggesting that cluster A with low m7G scores participates in the pathological mechanism of AD by regulating immune-related pathways. Number of B cells that produce immunoglobulins against Aβ is higher in patients with AD than in healthy individuals, which may affect plaque formation and disease progression [58]. Therapeutic depletion of B cells early during the onset of AD mice can reduce Aβ plaque burden and microglia activation [59]. Recent studies have indicated that Th1 and Th17 cells, which belong to distinct subclasses of CD4^+^ T cells, play a vital role in the pathogenesis of AD by triggering inflammatory responses in glial cells [60]. A previous study reported that exogenous monocytes from the bloodstream are recruited to the central nervous system, crossing the blood–brain barrier to potentially differentiate into brain-resident microglia [61]. NK cells, which can be abnormally activated in patients with AD, may also contribute to neuroimmune damage and cognitive impairment through excessive release of proinflammatory cytokines [62]. Increased neutrophil numbers have also been reported to be associated with increased Aβ deposition and cognitive deficits [63]. Alterations of these immune cells in the abovementioned studies were consistent with those observed in our study, the only difference being in the behavior of dendritic cells. Another study found a notable decrease in the number of myeloid dendritic cells in patients with AD compared with that in healthy individuals, a correlation linked to the progression of the disease [64]. The precise mechanism through which dendritic cells influence AD remains elusive and warrants further investigation.

Additionally, functional enrichment analysis showed that the DEGs of cluster A were predominantly enriched in brain-function–associated biological processes, such as ion transmembrane transport, synapse organization, and membrane potential regulation, as well as highly expressed in several hallmark gene sets, including the TYROBP causal network in microglia, TGF-β signaling pathway, and INF-γ signaling gene sets.

Based on these findings, we used three types of machine-learning algorithms to identify AD feature genes based on the DEGs in cluster A. Machine-learning algorithms can be applied to biomarker identification and elucidation of disease mechanisms [[65], [66], [67], [68]]. For feature-gene selection, we combined three machine-learning algorithms. Eventually, we identified five feature genes: *AEBP1*, *CARTPT, AK5, NPTX2,* and *COPG2IT1*. As a widely expressed transcriptional inhibitor, *AEBP1* plays a role in the regulation of inflammatory responses [69]. Its expression is elevated in AD hippocampi, particularly in neurons with neuritic plaque [70]. *CARTPT* can encode a neuropeptide involved in the regulation of appetite and satiety and may play a role in the association of obesity with AD [71]. *Ak5* is a purine metabolism gene involved in the phosphorylation of AMP to ADP and of dAMP to dATP [72]. *AK5* mRNA was observed to be significant downregulated in the cortex of AD stages V-VI [73]. *NPTX2* encodes a member of the neuronal pentraxins subfamily, which is involved in synaptic plasticity, synapse formation, and remodeling [74]. In AD, *NPTX2* is reduced in postmortem brain and in patient cerebrospinal fluid, and its concentration correlates with disease status, cognitive performance, and disease progression [75]. A recent study reported that *NPTX2* overexpression can ameliorate synapse loss in P301S mice [76]. *COPG2IT1* is an imprinted gene in the placenta, which is associated with infant neurobehavioral development [77]. Notably, the five-gene diagnostic model has high diagnostic value for AD, suggesting that *AEBP1*, *CARTPT, AK5, NPTX2,* and *COPG2IT1* can serve as biomarkers for AD. These feature genes have not been previously reported to be involved in the m7G methylation process. Nevertheless, the Pearson correlation analysis between the mRNA expression of feature genes and that of m7G regulator genes in the patients with AD in our study suggests that the stability and translation of these feature genes may be regulated by *NUDT11* and *QKI* in AD. This finding also offers potential targets to help elucidate the mechanism through which m7G methylation affects AD.

Additionally, experiments on AD mice, including qRT-PCR, IF, and IHC, have further confirmed the overexpression of *AEBP1* and downregulation of *CARTPT*, *NPTX2*, *COPG2IT1*, and *AK5* in AD, further strengthening the relevance of these biomarkers. To explore the cell-specific expression of the feature genes, we combined scRNA-seq data to analyze them and visualized the expression of the characterized genes in each cell type using violin plots. *AK5* was annotated in all cell types; *AEBP1* in astrocytes, PER/END cells, and T cells; and *CARTPT* and *NPTX2* were rarely annotated (Fig. 10B). *COPG2IT1* was not annotated in any of the seven cell types.

Some limitations of this study need to be stated. First, these genes have only been validated in mouse experiments and lack support from human samples. Second, the clinical information of AD patients, such as previous therapies, was incomplete. The potential mechanisms and pathways by which m7G methylation regulates AD immune infiltration need to be further explored, which is also the direction of our future research.

## 5 Conclusion

To our understanding, this study represents the first attempt to investigate the role of m7G methylation in AD. Two distinct AD subgroups were identified based on the m7G regulator genes. The two subgroups differed significantly in terms of clinical features, immune infiltration, and biological functions of AD, thus better elucidating the heterogeneity of AD patients. More importantly, we identified and experimentally validated five feature genes: *AEBP1*, *CARTPT, AK5, NPTX2,* and *COPG2IT1*. These genes were closely correlated with the prognosis of patients with AD and can potentially serve as biomarkers for the disease.

## Funding

This work was supported by grant from the 10.13039/501100001809National Natural Science Foundation of China (No. 82371270 and 81974200). Funding acquisition was supported by Xuebing C. and Yan X.

## Ethics approval and consent to participate

The animal study was reviewed and approved by the Experimental Animal Management Committee of Tongji Medical College of Huazhong University of Science and Technology (No. 3453).

## Consent for publication

Not applicable.

## Data availability statement

The datasets in this study can be free download in the GEO (https://www. ncbi. nlm. nih. gov/geo/) database.

## CRediT authorship contribution statement

**Piaopiao Lian:** Writing – original draft, Validation, Methodology, Data curation, Conceptualization. **Xing Cai:** Writing – original draft, Methodology, Conceptualization. **Cailin Wang:** Validation, Conceptualization. **Heng Zhai:** Writing – review & editing, Supervision. **Ke Liu:** Methodology. **Xiaoman Yang:** Methodology. **Yi Wu:** Methodology. **Zhuoran Ma:** Methodology. **Xuebing Cao:** Writing – review & editing, Supervision. **Yan Xu:** Writing – review & editing, Supervision, Project administration, Funding acquisition.

## Declaration of competing interest

The authors declare that they have no known competing financial interests or personal relationships that could have appeared to influence the work reported in this paper.

## Figures and Tables

**figs1:**
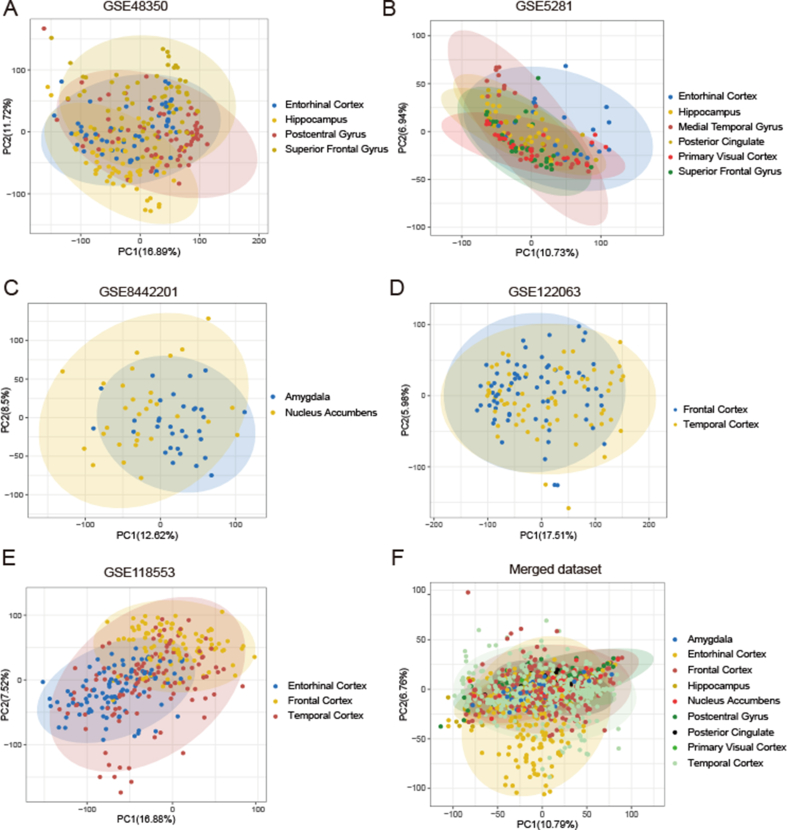


**figs2:**
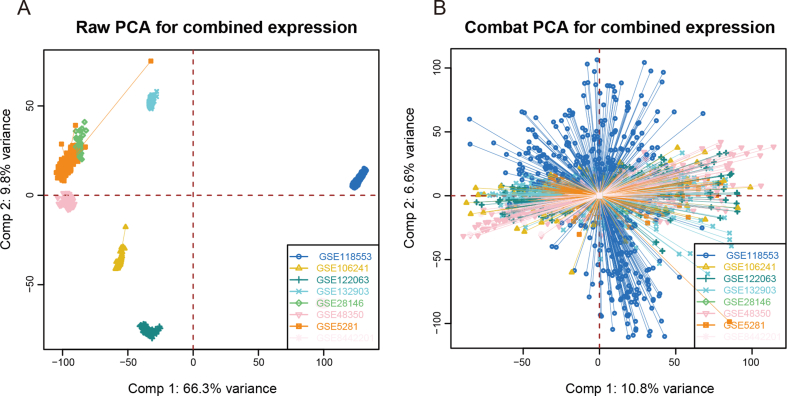


**figs3:**
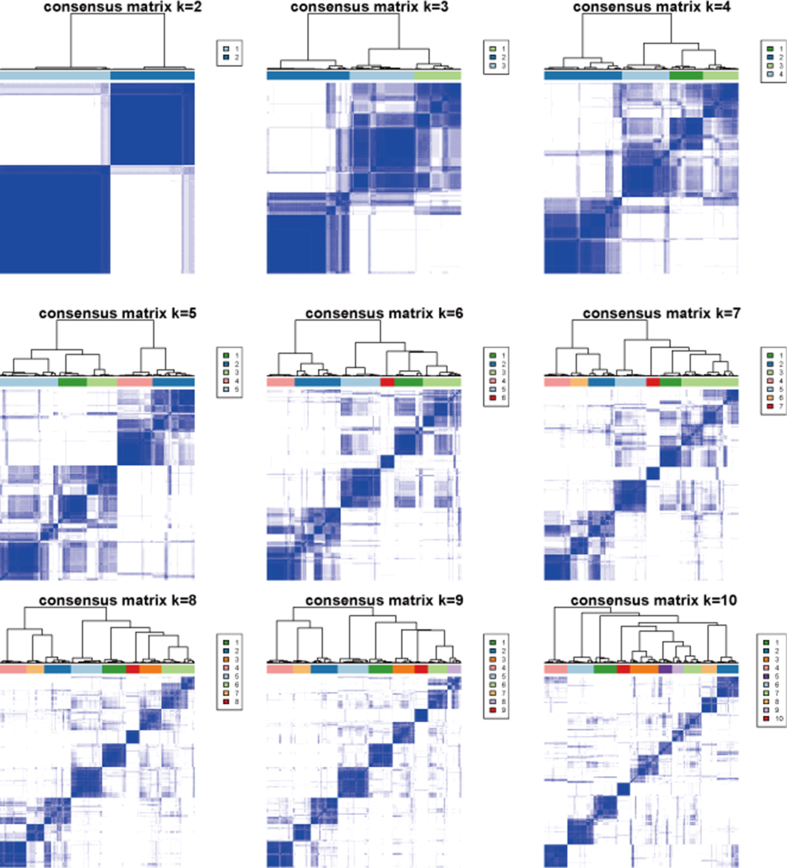


**figs4:**
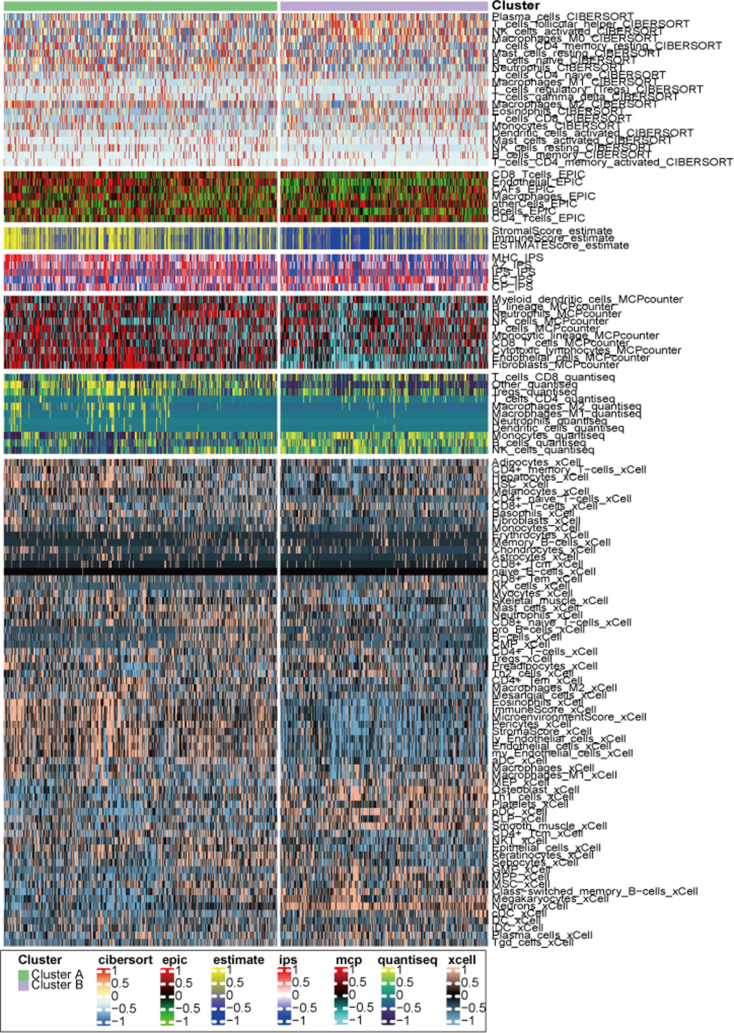


**figs5:**